# Editorial: A Microbial View of Central Nervous System Disorders: Interplay Between Microorganisms, Neuroinflammation and Behaviour

**DOI:** 10.3389/fimmu.2021.816227

**Published:** 2021-12-16

**Authors:** Davide Cossu, Robert O. Watson, Cinthia Farina

**Affiliations:** ^1^ Department of Neurology, Juntendo University, Tokyo, Japan; ^2^ Department of Biomedical Sciences, Sassari University, Sassari, Italy; ^3^ Department of Microbial Pathogenesis and Immunology, College of Medicine, Texas Agricultural and Mechanical (A&M) Health Science Center, Bryan, TX, United States; ^4^ Institute of Experimental Neurology and Division of Neuroscience, Istituto di Ricovero e Cura a Carattere Scientifico (IRCCS) San Raffaele Scientific Institute, Milan, Italy

**Keywords:** pathogens, multiple sclerosis, Alzheimer’s disease, neuroinflammation, Parkinson’s disease, microbiota, cognitive and psychiatric disorders

Various pathogens have been associated with initiation or exacerbation of neurological diseases such as Alzheimer’s disease and multiple sclerosis. This Research Topic collects knowledge acquired with animal and human studies about the complex host-pathogen interactions during neurological, cognitive, and psychiatric disorders.

Pathogens use unique molecular strategies altering the neuroimmune crosstalk, which initiate from the periphery or directly act in the central nervous system (CNS). Nucleotide-binding oligomerization domain-like receptors (NLRs) are a family of conserved cytosolic receptors that recognize both host- and pathogen-derived intracellular danger signals and play an important role in shaping host defenses and inflammation (1). The review by Zhang et al. focuses on NLR family caspase recruitment domain containing 5 (NLRC5), which has been shown to contribute to a wide variety of immune responses and has recently been connected to neuronal development and central nervous system (CNS) diseases. Here, the authors summarize recent progress on the structure, expression, and biology of NLRC5, its contribution to CNS function and involvement in neurological diseases.

Some studies suggest a link between infections and CNS disorders. In a review, Dow presents evidence supporting the hypothesis that *Mycobacterium avium* susp. *paratuberculosis* contributes to dysfunctional autophagy in Alzheimer-infected patients, which in turn may result in neurodegeneration or neurodegeneration-like symptoms. The fact that anti-mycobacterial agents such as rifampin appear to have a neuroprotective role not only in Alzheimer’s but also in Parkinson’s disease (2), supports the rationale for future investigations about the relationship between mycobacteria and neurodegenerative disorders. Further, Frau et al. summarize the role of three pathogens (EBV, HERV and *Mycobacterium avium* susp. *paratuberculosis*) in conferring risk for developing multiple sclerosis (MS) in Sardinia, a Mediterranean island characterized by peculiar genetic polymorphisms and high MS prevalence. Research evidence from studies with humans and animal models show that pathogens may manipulate gene expression in susceptible hosts, leading to immune dysregulation and tissue damage (3). Molecular mimicry seems to be the most accredited mechanism to explain their involvement in MS (3). Moreover, the synergistic interactions between multiple pathogens such as EBV and HERV may also play a role in the development and/or exacerbation of MS (Frau et al.)

Although progress has been made in understanding the underlying biology of MS, almost nothing is known about how this disease is triggered. It has been proposed that viral infections including herpes simplex type I (HSV-1) may contribute to precipitation and/or enhancement of MS (4). Duarte et al. leverage the widely used experimental autoimmune encephalomyelitis (EAE) model to study MS and evaluate the effect of asymptomatic brain infection by HSV-1 on the precipitation and severity of EAE in mice. Using both WT and mutant HSV-1, the authors find that HSV-1 infection is associated with more severe EAE in mice compared to uninfected control mice. Their findings support the idea that HSV-1 infection can accelerate and/or exacerbate EAE, which suggests a potential contribution of asymptomatic viral infection to the onset and severity of MS.

Recently, the relationship between chronic viral infection and MS has been complicated by the novel coronavirus, SARS-CoV-2, as its severity of infection heavily depends on host response to infection mirroring several aspects of MS pathobiology (Bellucci et al.). Here, Bellucci et al. take a close look over an 18-month period of SARS-CoV-2 pandemic through the lens of MS and discuss the neuroinflammatory and demyelinating mechanisms associated with COVID-19, review pathophysiological crosstalk between MS and SARS-CoV-2 infection, and investigate SARS-CoV-2 vaccination in the context of MS.

Neuroinflammation is a complex response to CNS injury, which involves the interaction between elements of innate and adaptive immunity. Among adaptive immune cells the regulatory T cells (Tregs) are a major determinant of immune tolerance. Schroeter et al. offers an overview of the role of Treg in various models of infection, the impact of pathogens on Treg number, phenotype and function, and the implications for autoimmune processes involved in multiple sclerosis. This review summarizes the results of clinical studies in MS where antimicrobial therapies (e.g. those directed to EBV, HERV or helminths) have been tested (Schroeter et al.). The evidence that certain microbes may lead to the expansion of Treg frequency opens to the possibility of therapeutic interventions reprogramming immune balance with pathogens or their derived products.

The gut-brain axis clearly plays a crucial role in neurological diseases, including MS (Parodi and Kerlero de Rosbo). Altered microbiota and increased intestinal permeability have been associated with pathogenesis of MS and its model, experimental autoimmune encephalomyelitis (EAE) (5). However the “cause and effect” of these connections remains unclear. In a review, Parodi and Kerlero de Rosbo sort through the evidence supporting two possible consequences of the gut-brain axis dysfunction in MS and EAE: a) a proinflammatory pro-inflammatory environment and “leaky” gut induced by dysbiosis contribute directly to MS Neuroinflammation; b) neuroinflammation precipitates intestinal inflammation as disease progresses.

Further, gut microbiota may impact mammalian development and behaviour as reported in studies using germ-free mice (6, 7). These observations have opened to the hypothesis that human behaviour may depend on the interaction between gut microbiota and the host. The review paper by Foster et al. summarizes the evidences about changes in gut microbiome associated with psychiatric conditions and underlines how major depressive disorders present with changes in several immune markers indicating a clear peripheral pro-inflammatory setting under this condition. Moreover, several studies indicate that exposure to various types of stress may alter immunity and gut microbiome and that, vice versa, the microbiome can regulate the impact of stress (6–9), pointing to future developments in the field of nutritional psychiatry.

## Author Contributions

All authors listed have made a substantial, direct, and intellectual contribution to the work and approved it for publication.

## Funding

Davide Cossu’s research is supported by JSPS KAKENHI (grant number: JP 20K16468). Cinthia Farina's research is supported by Italian Ministry for Health (Ricerca Corrente and RF-2018-12367731), FISM (Fondazione Italiana Sclerosi Multipla, grant number 2016/R/14) and cofinanced with the 5 per mille public funding.

## Conflict of Interest

The authors declare that the research was conducted in the absence of any commercial or financial relationships that could be construed as a potential conflict of interest.

## Publisher’s Note

All claims expressed in this article are solely those of the authors and do not necessarily represent those of their affiliated organizations, or those of the publisher, the editors and the reviewers. Any product that may be evaluated in this article, or claim that may be made by its manufacturer, is not guaranteed or endorsed by the publisher.
